# From Grotthuss Transfer to Conductivity: Machine Learning Molecular Dynamics of Aqueous KOH

**DOI:** 10.1021/acs.jpcb.5c03199

**Published:** 2025-06-09

**Authors:** V. Jelle Lagerweij, Sana Bougueroua, Parsa Habibi, Poulumi Dey, Marie-Pierre Gaigeot, Othonas A. Moultos, Thijs J. H. Vlugt

**Affiliations:** † Engineering Thermodynamics, Process and Energy Department, Faculty of Mechanical Engineering, 2860 Delft University of Technology, Leeghwaterstraat 39, Delft 2628CB, The Netherlands; ‡ 27048 Université Paris-Saclay, Univ Evry, CY Cergy Paris Université, CNRS, LAMBE, Evry-Courcouronnes 91025, France; ¶ Department of Materials Science and Engineering, Faculty of Mechanical Engineering, 2860 Delft University of Technology, 2628CD Delft, The Netherlands; § Institut Universitaire de France (IUF), 75005 Paris, France

## Abstract

Accurate conductivity predictions of KOH­(aq) are crucial for electrolysis applications. OH^–^ is transferred in water by the Grotthuss transfer mechanism, thereby increasing its mobility compared to that of other ions. Classical and ab initio molecular dynamics struggle to capture this enhanced mobility due to limitations in computational costs or in capturing chemical reactions. Most studies to date have provided only qualitative descriptions of the structure during Grotthuss transfer, without quantitative results for the transfer rate and the resulting transport properties. Here, machine learning molecular dynamics is used to investigate 50,000 transfer events. Analysis confirmed earlier works that Grotthuss transfer requires a reduction in accepted and a slight increase in donated hydrogen bonds to the hydroxide, indicating that hydrogen-bond rearrangements are rate-limiting. The computed self-diffusion coefficients and electrical conductivities are consistent with experiments for a wide temperature range, outperforming classical interatomic force fields and earlier AIMD simulations.

## Graphical abstract

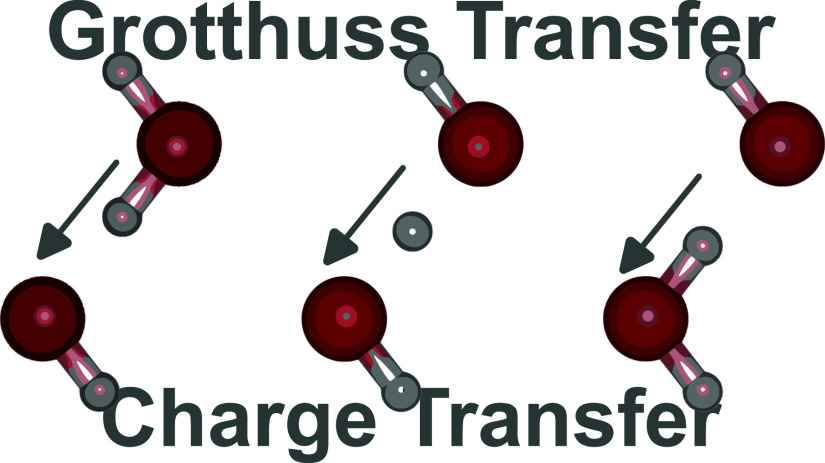

## Introduction

Aqueous potassium hydroxide (KOH­(aq)) has a remarkably high electrical conductivity compared to other aqueous salts.^1,2^ This is ideal for applications in which minimizing conductive losses in electrolytes is crucial. Key applications are alkaline water electrolysis,^3–5^ electrochemical CO_2_ reduction,^6,7^ capacitors,^8–10^ and batteries.^11^ The high electrical conductivity of aqueous hydroxide mixtures (OH^–^) stems from the Grotthuss transfer mechanism (i.e., proton transfer, proton hopping).^12,13^ This mechanism is a molecular identity switch of OH^–^ ions with water (see Figure 1), which enhances the mobility of OH^–^, resulting in higher self-diffusivities and electrical conductivities.^12,13^ Although the Grotthuss transfer has been investigated qualitatively with ab initio molecular dynamics (AIMD),^13–21^ there is limited understanding of its quantitative effects on self-diffusion and electrical conductivity of OH^–^. This is due to the computational costs of AIMD, limiting this method to small system sizes and short simulation time scales.

**Figure 1:**
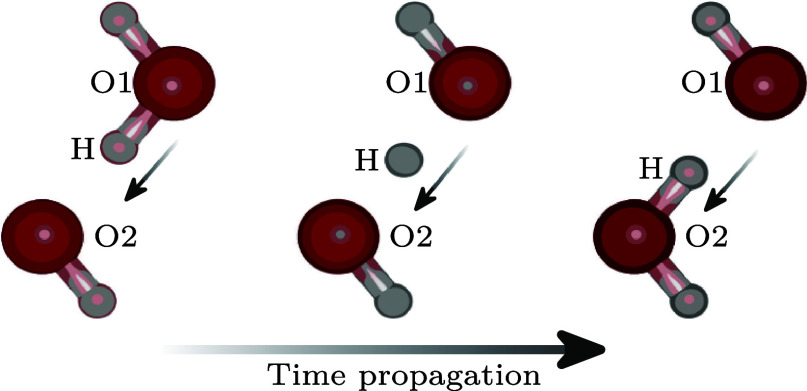
Schematic description of Grotthuss transfer, where the OH^–^ takes a hydrogen of a H_2_O molecule. Before the transfer event, oxygen 1 (O1) and the center hydrogen (H) are part of the H_2_O molecule. After the reaction, O1 becomes part of the OH^–^ molecule, and H is now chemically bonded to O2. As this reaction is an identity switch, no change in concentration occurs.

Here, we study the multiscale effect of the Grotthuss transfer in KOH­(aq) with a machine learning force field (MLFF) trained on ab initio data, thereby overcoming the time and length scale limitations of AIMD. In addition to light water, simulations with heavier hydrogen isotopes provide insights into isotope effects on the transfer mechanism. For the first time, simulated electrical conductivities match experimental values within 5% accuracy. Classical MD simulations are also performed to indicate the relevance of capturing the Grotthuss transfer. AIMD and machine learning molecular dynamics (MLMD) simulations are compared to ensure that the MLFF is fitted accurately. The graph theory postprocessing tool GaTewAY^22–28^ was used to compare the hydrogen bonding of OH^–^ in reactive and nonreactive configurations. Statistical analysis of more than 50,000 Grotthuss events revealed that the OH^–^ loses a hydrogen bond just before a transfer event. The hydration of OH^–^ is similar to that of water during Grotthuss events. Both the reaction energy barrier and the time between Grotthuss transfer events determined here are in agreement with the experimental results^29,30^ of hydrogen-bonding rearrangements. This confirms that the loss of the hydrogen bond of OH^–^ is the rate-limiting step of Grotthuss transfer at low concentrations.^31^

Previous simulation studies provided molecular insights into Grotthuss transfer. AIMD simulations performed by Tuckerman et al.^14–18^ and later by Agmon et al.^19,20^ revealed how the Grotthuss transfer of OH^–^ differs from the corresponding process for H_3_O^+^ in aqueous solutions. These studies explain qualitatively why H_3_O^+^ diffuses faster than OH^–^, while a few self-diffusion coefficients of OH^–^ (*D*_OH^−^_) are reported, as well. Nevertheless, the reported *D*_OH^–^_ had high statistical uncertainty due to short simulation times (≈10 ps). Long (400 ps) AIMD simulations of OH^–^ have been reported by Muñoz-Santiburcio.^21^ Still, these simulations remain too short for the mean squared displacements of individual ions to reach the square of half the box size.^21^ This check is necessary to determine whether the simulation time is sufficient for self-diffusion computations.^32^

Classical force fields (FFs) cannot model chemical reactions, such as the Grotthuss transfer, and fail to capture *D*_OH^−^_. For example, the DFF/OH^–^ FF,^33^ a classical FF that captures other properties accurately, underestimates the experimental *D*_OH^−^_ by a factor of ca. 2. FFs capable of modeling reactions, such as ReaxFF^34,35^ or MOBHY,^36^ do capture Grotthuss transfer qualitatively. Parameterizing these FFs involves numerous assumptions. This leads to significant differences between simulated properties and experiments.^37^ For example, ReaxFF overestimates the OH^–^ self-diffusion coefficient by a factor of ca. 2. With ML, two- and three-body interactions relying on generic interatomic functionals (unlike the fixed forms used in classical or reactive FFs) can be trained using ab initio results on specific system snapshots. A properly trained MLFF provides interatomic forces close to AIMD at only slightly higher computational costs, comparable to classical FFs.^38^ Studies using MLFFs have contributed new insights into diverse systems, including pure water,^39–41^ electrolytes,^42–44^ and reactive mixtures.^45,46^ MLFFs have sparked renewed interest in Grotthuss transfer, as both the relevant time scales and the level of accuracy required to study this mechanism have now become accessible. Simulations of hydronium transfer^47^ and of NaOH­(aq) by Hellström and Behler et al.^31,48–50^ used MLFFs to investigate the concentration dependence of Grotthuss transfer, the role of nuclear quantum effects (NQEs),^47,49^ as well as the role that Grotthuss transfer and NQEs play in water self-ionization.^51^

## Computational Details

VASP 6.4.3 ^42,52–58^ was used for all ab initio and ML simulations. Periodic boundary conditions were set in all directions. The Nosé–Hoover thermostat^59,60^ (lattice mass of 5), the Verlet time integration scheme^61^ (time step of 0.5 fs), and the RPBE-D3 ^62–65^ density functional were applied. NQEs were not considered here as we aim to show that simple on-the-fly machine learning simulations capture the electrical conductivity accurately. Including NQEs would increase complexity and computational costs. RPBE-D3 without NQEs has shown excellent performance in predicting water structures^66^ and dynamics,^21^ as well as hydroxide diffusion.^21,49^ Including NQEs with this density functional overestimates hydroxide diffusion.^49^ This may be due to error cancellation, but it significantly simplifies the simulation approach. The DFT simulations involved a single k-point, 550 eV energy cutoff, 0.3 eV Gaussian smearing width, and a 0.01 meV convergence limit for the self-consistency cycles. Training of the MLFF was conducted at the experimental density at 72 °C.^2^ The system consisted of 110 H_2_O molecules and 12 KOH molecules. The initial configuration was created using fftool (V1.2.1)^67^ and PACKMOL (V20.3.1).^68^ Four on-the-fly ML simulations with an *NPT* temperature ramp from −20 to 72 °C were used to select training snapshots to retrain a final fast MLFF using the SVD solver in VASP. The hyperparameters of the MLFF were optimized, with two- and three-body cutoffs of 13 and 4 Å, respectively. The numbers of basis functions of two- and three-body interactions of the VASP MLFF were 12 and 8, respectively.

The MLMD production simulations were performed at a lower concentration than the training (ca. 0.5 mol of KOH per kg of H_2_O). The simulation box contained 110 H_2_O molecules and 1 KOH molecule. MLMD simulations of the three hydrogen isotopes, H, D, and T, were performed using the same box sizes, FF, and other simulation settings. *NVT* simulations were conducted at experimental densities of KOH (aq)^2^ in the range of 15–65 °C at 10 °C intervals, using the Nosé–Hoover thermostat. Diffusion coefficients are not affected by this thermostat for the simulated system sizes.^21,32^ The periodic boundary conditions and time step size remained consistent with the training phase. Six independent simulations were performed for each state point to obtain statistical uncertainties, which are reported as twice the standard error of the mean. The simulations consisted of a 20 ps equilibration followed by a 1000 ps production run. AIMD production runs used the same ab initio settings as the training phase, with simulation boxes identical to those in the MLMD simulations. These were only performed at 15 °C and consisted of 3.5 ps equilibration and 3.5 ps production runs; 120 AIMD simulations were performed to accurately sample the radial distribution functions of the ions and the intramolecular shape of the water molecules. Classical MD simulations were performed using LAMMPS (Mar2018)^69,70^ with the OCTP plugin^71^ at the same thermodynamic state points as the MLMD simulations. The coupling time of the Nosé–Hoover thermostat^59,60^ was 100 fs, and the simulation box contained 1100 H_2_O and 10 KOH molecules. The TIP4*P*/2005 water^72^ and DFF/OH^–^ ^33^ FFs were used. The six simulations per state point consisted of over 1 ns of equilibration and 25 ns of production each. See the Supporting Information for detailed simulation settings.

## Results and Discussion

The accuracy of the MLFF was assessed by comparing the forces and radial distribution functions of MLMD with AIMD. The total force on each atom was computed for random equilibrated configurations with both methods; see Figure 2a. The test configurations were at the same concentration as those of the production runs (0.5 mol of KOH per kg of H_2_O). This is significantly below that of the training data (6 mol of KOH per kg of H_2_O). The accuracy of the MLFF in predicting intramolecular forces is assessed by comparing the water angle and bond length from MLMD and AIMD simulations. At 15 °C, MLMD simulations yield *r*_OH_ = 0.97(1)­Å and an angle of θ_HOH_ = 104.8(5)°. AIMD simulations predict *r*_OH_ = 0.98(1)­Å and θ_HOH_ = 104.9(5)°. The intermolecular RDFs of K^+^ and OH^–^, illustrated in Figure 2b,c, indicate that AIMD and MLMD result in the same ion hydration. The hydration numbers of OH^–^ are 5.57(9) and 5.34(9) for MLMD and AIMD, respectively, matching the experimentally determined value 5.5(5)^73^ and the simulation result 5.8.^15^ The hydration numbers of K^+^ are 7.47(4) and 7.9(1) for MLMD and AIMD, respectively, which is close to the value 7.1(4) computed by Ikeda et al.^74^ Overall, the correlation between atomic forces and the transfer of structural properties from AIMD to MLMD validate the MLFF.

**Figure 2:**
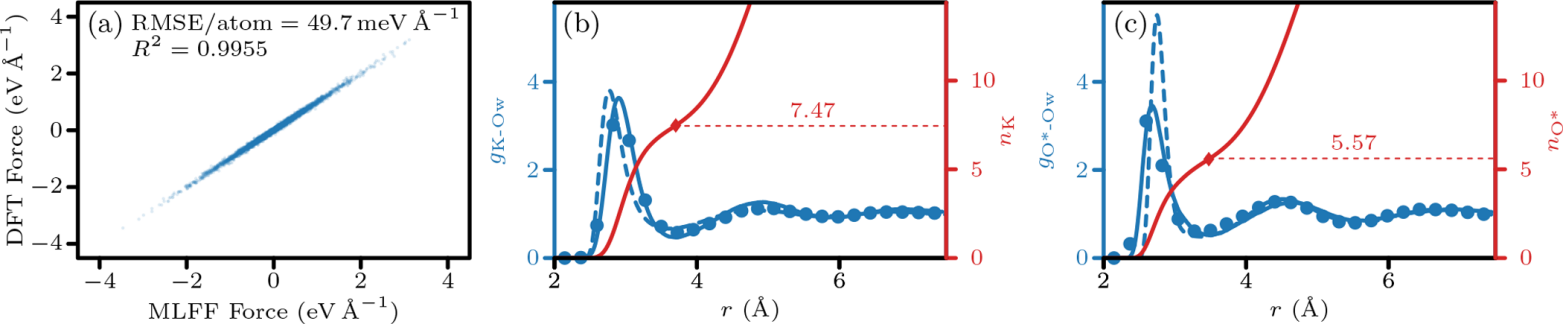
(a) Parity between forces predicted by DFT versus the MLFF. (b) and (c) Radial distribution function of K^+^ and the oxygen of the OH^–^ (O*) with the oxygen of water molecules (Ow), respectively. The continuous blue lines indicate the *g*(*r*) of MLMD, the blue circles indicate AIMD, and the dashed blue lines indicate classical MD. The radius depending on the hydration number is the red line, where the diamond indicates the hydration number at the minimum of *g*(*r*).

The 1 ns MLMD simulation times made in-depth statistical analysis of the Grotthuss transfer events possible, as more than 6500 transfer events per state point were captured. Tuckerman et al.^17^ already observed that there are two relevant hydration modes to the Grotthuss transfer of OH^–^. Our simulations also revealed two hydration modes: (1) a reactive hydration mode, where the proton can be passed between a H_2_O and OH^–^, and (2) a nonreactive hydration mode, where the hydrogen-bonding network is restructured. In a reactive configuration, the proton bounces back and forth quickly between the same two oxygen atoms multiple times. This does not add significantly to the transport of OH^–^, as the oxygen atoms involved do not travel far in this time span. Charge transport is effective only when the Grotthuss transfer occurs with a new H_2_O molecule, for which a restructured hydrogen-bonding network is necessary. This also indicates two separate time scales: (1) the time scale of the reactive mode, of 0.01–0.1 ps, and (2) a long time scale during the nonreactive mode, of 0.5–10 ps. To determine the effective lifetimes of OH^–^, the stable point picture approach is used.^75,76^ We sampled the probability of a stable OH^–^ turning into stable water^18,49^ within a time interval Δ*t* using the Lionanalysis software.^49^ Using stable states for the start and end points of the reaction analysis excludes the proton bouncing back and forth in an unstable state from the statistics, so only effective transfer events are sampled. The resulting correlations, shown in Figure 3a, are fitted to *a* exp­(−Δ*t*τ_1_^–1^) + (1 – *a*) exp­(−Δ*t*τ_2_^–1^), from which the lifetime is determined with τ = *a*τ_1_ + (1 – *a*)­τ_2_. Table 1 reports τ values for the simulation temperatures and hydrogen isotopes. The reaction energy barrier (*E*_barr_) and pre-exponential factor (*A*), determined from the Arrhenius temperature dependence of τ, are reported as well. Isotopes have approximately the same electron distribution and thus share the same interatomic potential, although the dynamics differ due to the change in atomic mass. This leads to no significant changes in *E*_barr_ and a reduction of *A*, which can be observed from Table 1. Changes in *E*_barr_ may be expected if NQEs were concidered^49,77^ since such techniques capture the differences between nuclei. The effective hydroxide lifetimes and the reaction energy barrier computed from the light water simulations are slightly larger than found in other studies,^29,30^ which might indicate that effective Grotthuss transfer is connected to long hydrogen-bond lifetimes.^78^

**Table 1:** Effective OH^–^ Lifetimes τ Computed with the Stable Point Picture Approach,^75,76^ Reaction Energy Barrier *E*_barr_, and Pre-Exponential Component *A* of the Arrhenius Equation for All Simulated Hydrogen Isotopes^a^

	*T* (°C)	15	25	35	45	55	65
H_2_O + KOH	τ (ps)	5.3(1)	4.1(1)	3.8(2)	3.4(1)	3.3(1)	2.89(1)
	*E*_barr_ (kJ mol^–1^)	9.4(9)					
	*A* (ps^–1^)	10(3)					
							
D_2_O + KOD	τ (ps)	5.5(1)	4.8(3)	4.6(5)	4.0(1)	3.48(4)	3.3(1)
	*E*_barr_ (kJ mol^–1^)	9.3(3)					
	*A* (ps^–1^)	9(1)					
							
T_2_O + KOT	τ (ps)	6.25(6)	5.3(1)	4.3(6)	4.31(3)	3.8(5)	3.5(1)
	*E*_barr_ (kJ mol^–1^)	9.7(5)					
	*A* (ps^–1^)	9(2)					

a The values in the parentheses are twice the standard deviation of the mean in the least-significant digits.

**Figure 3:**
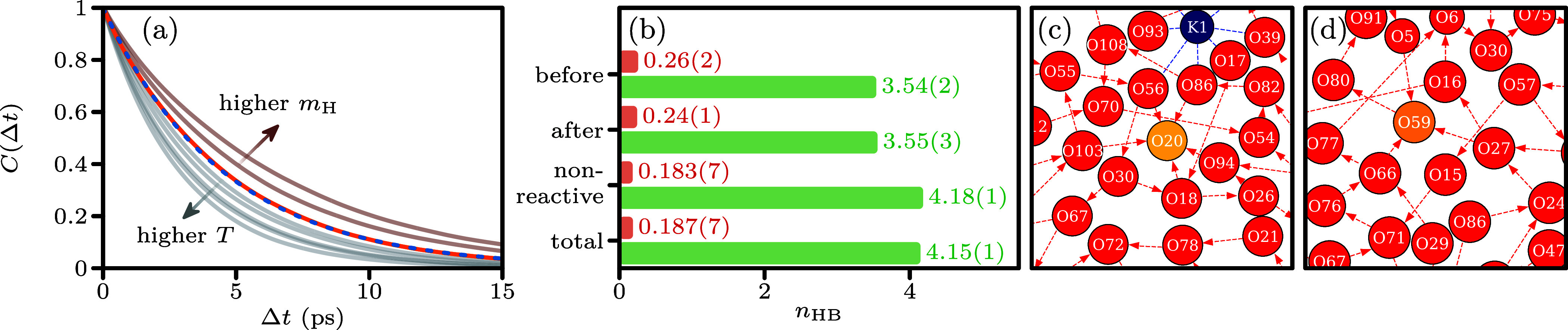
(a) Time correlation functions *C*(Δ*t*) from which the OH^–^ lifetimes τ are computed using the stable state picture.^75,76^ The orange curve shows simulation results at 15 °C, and the dashed blue line is fitted to *A* exp­(−Δ*t*τ_1_^–1^) + (1 – *A*) exp­(−Δ*t*τ_2_^–1^). The gray lines below the orange curve indicate the other temperatures (25–65 °C), and the light-brown lines above the orange curve are simulation results with heavy water at 15 °C, with *m*_H_ = 2, 3 u. (b) Bar chart of the average number of hydrogen bonds in the reactive configuration (50 fs before and after Grotthuss transfer) and the nonreactive hydration mode. The total average hydrogen-bond number (*n*_HB_) is indicated, as well. We separated the results in donated (red bars) and accepted (green bars) hydrogen bonds. (c) and (d) Typical GaTewAY^22–28^ graphs of the hydrogen-bonding network in the nonreactive and reactive hydration modes, respectively. The color indicates the molecule type: K^+^ is blue, OH^–^ is yellow, and H_2_O is red. The red arrows between the molecules indicate the direction of the hydrogen bonds (from donor to acceptor). The blue dashed lines indicate the ionic interactions between K^+^ and the water.

The local environment of OH^–^ was investigated using the total number of hydrogen bonds. The hydrogen bonding of the reactive and nonreactive modes is compared in Figure 3b. We separated the MLMD trajectories in three categories: (1) all time steps in the 50 fs window before a Grotthuss transfer event; (2) the time steps in the same window after a transfer event; and (3) all time steps not included in (1) or (2). Categories (1) and (2) are in the reactive mode, and category (3) is in the nonreactive mode. To avoid double-counting of configurations, the interval is split evenly between (1) and (2) if two consecutive reactions occurred within 100 fs. On average, 4.37(2) hydrogen bonds were connected to the OH^–^ in the nonreactive mode. An example of a nonreactive hydrogen-bonding network is visualized in Figure 3c, illustrating the OH^–^ accepting five hydrogen bonds. OH^–^ in the nonreactive mode accepts many more hydrogen bonds than water, which generally forms 3–4 hydrogen bonds. There were significantly fewer hydrogen bonds in the reactive mode: 3.80(3) before and 3.80(4) after Grotthuss transfer events. This is illustrated in Figure 3d, with the OH^–^ donating one and accepting three hydrogen bonds. The reduction in hydrogen bonding when the OH^–^ transitions from the nonreactive to the reactive mode suggests that Grotthuss transfer can occur only if OH^–^ is hydrated in a manner that also accommodates a H_2_O. The hydrogen bonds are categorized into accepted and donated bonds for a more detailed analysis (Figure 3b). This reveals a significant reduction in accepted hydrogen bonds and a slight increase in donated hydrogen bonds. These findings are consistent with those reported by Tuckerman et al.,^17^ although our results indicate a smaller increase in donated hydrogen bonds.

The self-diffusion coefficients of the K^+^ (*D*_K^+^_) and OH^–^ were computed and compared with classical MD simulations.^33^ Minor differences in *D*_K^+^_ were observed between MLMD and classical MD simulations, as shown in Figure 4a. The significant differences for *D*_OH^−^_ (Figure 4b) highlight the effectiveness of MLFF in capturing the Grotthuss transfer. The electrical conductivity of the overall mixture, shown in Figure 4c, was calculated with the Nernst–Einstein equation. Although the Nernst–Einstein equation does not include ion–ion correlations, it can be corrected for its known finite-size effects.^80,81^ Such corrections are not yet available for direct computations of electrical conductivity using Green–Kubo or Einstein–Helfand equations. Finite-size effects are more significant than ion–ion correlations at the simulated system sizes and concentrations. Direct calculations of electrical conductivities without finite-size corrections are reported in the Supporting Information. The MLMD results fit experimental data in Gilliam et al.,^2^ while the classical MD simulations underestimate the electrical conductivity significantly. Our results show closer agreement with experiments compared to previous MLMD simulations investigating the concentration-dependent electrical conductivity in NaOH­(aq).^50^ The difference likely arises from our MLFF being optimized for a single concentration, whereas optimizing across multiple concentrations can improve transferability, introduces added complexity, and may compromise accuracy.

**Figure 4:**
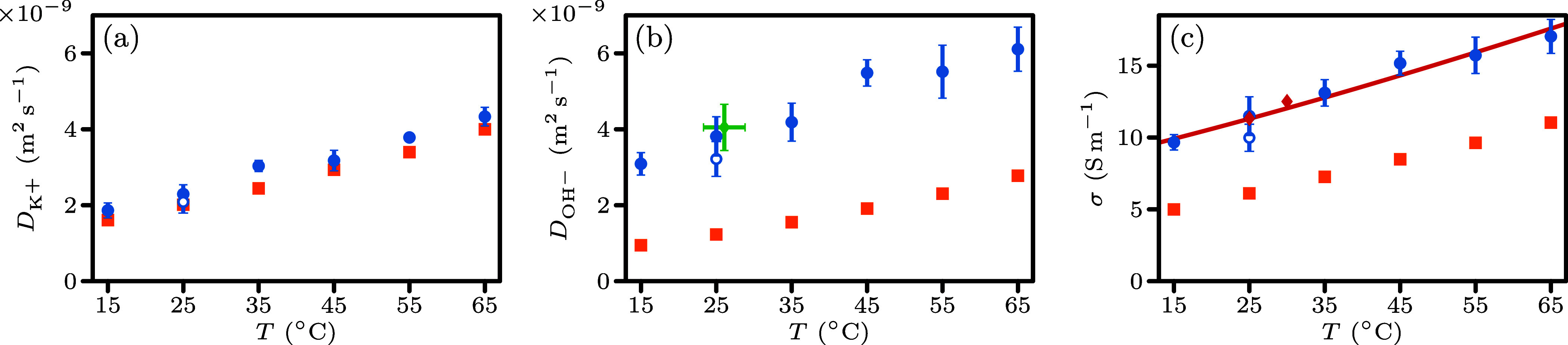
(a) and (b) Self-diffusion coefficients of K^+^ and OH^–^, respectively. (c) Electrical conductivities of the KOH­(aq) mixture. In all subfigures, the closed blue circles are the MLMD results of light water, the open blue circles are the MLMD results of heavy water (*m*_H_ = 2 u), and the orange squares are the classical MD data points. The green diamond (b) is the AIMD result by Muñoz-Santiburcio. The red diamond and curve (c) represent experimental results of electrical conductivities^79^ and an experimental fit curve,^2^ respectively.

## Conclusions

This study investigated changes in OH^–^ hydration during Grotthuss transfer, the self-diffusion coefficients of the ions, and the electrical conductivity. We rigorously trained and tested our MLFF, which resulted in excellent structure properties compared with the AIMD simulations. The results quantitatively confirm the transfer mechanism at low concentrations, introduced by Tuckerman et al.^14–18^ and found as well by Hellström and Behler et al.^31,48,49^ The computed electrical conductivity, which is of special relevance for this mixture, matches experiments within 5% for the first time. The MLMD techniques provide a method to investigate potential additives, such as cheotropic salts, that destabilize the hydrogen-bonding structure of OH^–^. This would lead to an increased Grotthuss transfer and higher electrical conductivity. Investigating the intricate effects of such additives on Grotthuss transfer would require a more detailed approach, for example, meta-GGA or hybrid density functionals combined with path integral methods to account for NQEs. These density functionals and simulation techniques have even higher computational costs. In parallel, graph theory analysis tools such as GaTewAY could analyze the hydrogen-bond network beyond the first hydration shell, which may offer insights into how longer-range structural organization influences Grotthuss transfer. This makes MLFFs especially relevant as these enable the use of such high-accuracy methods at fractions of the computational costs. This enables investigating the tuning of chemical properties by molecular modifications, which is possible only when simulations are both highly accurate and computationally feasible.

## Supplementary Material



## Notes

Simulation settings of all AIMD, MLMD, and classical MD simulations; mathematical descriptions of the applied postprocessing methods; and tabulated simulation results (PDF)
