# Supplementary material for: From Grotthuss Transfer to Conductivity: Machine Learning Molecular Dynamics of Aqueous KOH
Source: J Phys Chem B. 2025 Jun 9;129(24):6093–9. doi: 10.1021/acs.jpcb.5c03199 (PMC12183714; doi:10.1021/acs.jpcb.5c03199)
Supplement: Supplementary file 1 [file jp5c03199_si_001.pdf]

Supporting Information: From Grotthuss Transfer to Conductivity: Machine Learning Molecular Dynamics of Aqueous KOH

V.J. Lagerweij,[†] S. Bougueroua,[‡] P. Habibi,[†] P. Dey,[¶] M.-P. Gaigeot,[‡] O.A. Moultos,[†] and T.J.H. Vlugt[*,†]

†*Engineering Thermodynamics, Process & Energy Department, Faculty of Mechanical Engineering, Delft University of Technology, Leeghwaterstraat 39, Delft 2628CB, The Netherlands*

‡*Université Paris-Saclay, Univ Evry, CY Cergy Paris Université, CNRS, LAMBE, Evry-Courcouronnes 91025, France*

¶*Department of Materials Science and Engineering, Faculty of Mechanical Engineering, Delft University of Technology, 2628CD Delft, the Netherlands*

§*Institut Universitaire de France (IUF), 75005 Paris, France*

E-mail: t.j.h.vlugt@tudelft.nl

In this Supporting Information, we list all simulations performed for this publication, the settings of these simulations, as well as the post-processing steps. All results presented in the main document are reproducible with the following information. The mean squared displacements of the ions are illustrated as well to show how the self-diffusion coefficients are calculated.

1. Detailed Simulation Settings

For this work, five different simulation setups are used: (1) MLFF selection (see Table S1) is used to select suitable ML snapshots with on-the-fly ML.[S1–S4] The MLFF is refitted from the ab initio solutions of all selected configurations of the MLFF selection simulations; (2) The MLFF is fully utilized in the MLMD simulations (Table S2), producing the main results of the manuscript; (3) The plain AIMD simulations (Table S3) validate the MLFF performance; and (4) the classical MD simulations (Table S4) are used to compare self-diffusion coefficients and electrical conductivities.

Table S1: Simulation setting for the MLFF selection of configurations with on-the-fly ML.[S1–S4] These simulations started from well-equilibrated configurations.

**General**	
Software	VASP 6.4.3[S1–S8]
Number of Simulations/state point	5
**State points**	
Concentration (mol KOH/kg H_2O)	6
Number H_2O	110
Number K^+ and OH^-	12
Temperature (K)	Continuous increase 200 to 500
Density ($kg\,m^{-3}$)	1240.0
Box size (Å)	15.263
**Force and Energy Calculations**	
PAW Basissets	PBE H_h_GW, PBE O_GW_new, PBE K_sv_GW
GGA method	RPBE-D3[S9–S12]
Number of k-points	1 by 1 by 1 regular Gamma mesh
plane-wave energy cutoff (eV)	550
Gaussian smearing width (eV)	0.3
Convergence criterion (eV)	1×10^{-5}
MLFF type	On-the-fly
Cutoff 2-body (Å)	6
Number basis functions 2-body	10
Cutoff 3-body (Å)	6
Number basis functions 3-body	8
**Dynamics**	
Time step size (fs)	0.5
Number of time steps	10 000
Integrator	*NVT* Nosé-Hoover Verlet
Thermostat coupling strength	5

Table S2: Simulation settings of the MLMD simulations. These simulations started from AIMD equilibrated configurations. The densities and box sizes are computed from the temperatures using experimental relations determined by Gilliam et al.[S13]. The heavy water simulations with hydrogen isotopes of 2 (D), and 3 u (T) are performed at identical temperatures and box sizes with the same simulation settings. Only the mass of the hydrogen atoms, and thus the density, has been changed.

**General**	
Software	VASP 6.4.3[S1–S8]
Number of Simulations/state point	6
**State points**	
Concentration (mol KOH/kg H_2O)	0.5
Number H_2O	110
Number K^+ and OH^-	12
Temperatures (K)	288.15, 298.15, 308.15, 318.15, 328.15, and 338.15
Densities ($kg\,m^{-3}$)	1023.0, 1020.9, 1017.9, 1013.9, 1009.3, and 1004.2
Box sizes (Å)	14.900, 14.910, 14.925, 14.944, 14.967, and 14.992
**Force and Energy Calculations**	
MLFF type	Refitted with SVD solver no on-the-fly error estimation
Cutoff 2-body (Å)	13
Number basis functions 2-body	12
Cutoff 3-body (Å)	4
Number basis functions 3-body	8
**Dynamics**	
Time step size (fs)	0.5
Number of time steps	2 000 000
Integrator	*NVT* Nosé-Hoover Verlet
Thermostat coupling strength	5

Table S3: Simulation settings of the AIMD simulations used to validate the MLFF. These simulations started from AIMD equilibrated configurations. The density and box size are computed from the temperature using experimental relations determined by Gilliam et al.[S13].

**General**	
Software	VASP 6.4.3[S1–S8]
Number of Simulations/state point	120
**State points**	
Concentration (mol KOH/kg H_2O)	0.5
Number H_2O	110
Number K^+ and OH^-	1
Temperature (K)	288.15
Density ($kg\,m^{-3}$)	1023.0
Box size (Å)	14.900
**Force and Energy Calculations**	
PAW basis sets	PBE H_h_GW, PBE O_GW_new, PBE K_sv_GW
GGA method	RPBE-D3[S9–S12]
Number k-points	1 by 1 by 1 regular Gamma mesh
plane-wave energy cutoff (eV)	550
plane-wave energy cutoff (eV)	550
Gaussian smearing width (eV)	0.3
Convergence criterion (eV)	1×10^{-5}
**Dynamics**	
Time step size (fs)	0.5
Number of time steps	7000
Integrator	*NVT* Nosé-Hoover Verlet
Thermostat coupling strength	5

Table S4: Simulation settings of the classical MD simulations. These simulations started from random configurations created with fftool (V1.2.1)[S14] and PACKMOL (V20.3.1).[S15] The equilibration of the configurations was performed in NPT. The listed box sizes and densities are the results of the NPT equilibration.

**General**	
Software	LAMMPS (Mar2018)[S16,S17] with the OCTP plugin[S18]
Number of Simulations/state point	6
**State points**	
Concentration mol KOH/kg H_2O	0.5
Number H_2O	1100
Number K^+ and OH^-	10
Pressures (atm)	1.00
Temperatures (K)	288.15, 298.15, 308.15, 318.15, 328.15, and 338.15
Resulting densities (kg/m^3)	1024.7, 1022.0, 1018.3, 1013.8, 1008.7, and 1003.0
Resulting Box sizes (Å)	32.082, 32.111, 32.149, 32.197, 32.251, and 32.312
**Force and Energy Calculations**	
Force Field	TIP4P/2005[S19] and DFF/OH^-[S20]
LJ cutoff (Å)	10, with LJ tail corrections for energy and pressure
Long range interactions	PPPM with a relative accuracy of 1.0×10^{-5}
NPT **Equilibration Dynamics**	
Time step size (fs)	1
Number of time steps	1 000 000
Integrator	NPT Nosé-Hoover Verlet
Thermostat coupling time (fs)	100
Barostat coupling time (fs)	1000
NVT **Production Dynamics**	
Time step size (fs)	1
Number of time steps	25 000 000
Integrator	NVT Nosé-Hoover Verlet
Thermostat coupling time (fs)	100

2. Structural Properties

Strucural properties were computed to compare MLMD and AIMD simulations. The radial distribution function between molecules a and b ($g_{ab}(r)$) was calculated with the potential mean force technique:[S21,S22]

$$g_{ab}(r) = \frac{1}{8\pi k_\mathrm{B}T}\frac{V}{N_aN_b}\left\langle \sum_{i=1}^{N_a}\sum_{j=1}^{N_b}\frac{(\mathbf{F_i}-\mathbf{F_j})\cdot(\mathbf{r_j}-\mathbf{r_i})}{r_{ij}^3}\theta(r-r_{ij})\right\rangle, \tag{1}$$

where k_B is the Boltzmann constant, T the absolute temperature, N_i the total number of particles of species i, and V volume of the system. $\mathbf{F}$ is the total force on particles i and j, and $\mathbf{r}$ the position vector of particles i and j. r_{ij} is the Euclidean distance between the two particles, and θ indicates the Heaviside step function. This technique samples more efficiently than the traditional binning method,[S21,S22] which is especially relevant for the short AIMD simulations. n_{ab}, the coordination number between molecules a and b in the system was determined using the radial distribution function

$$n_{ab} = \frac{4\pi N_b}{V}\int_{r=0}^{r_\mathrm{shell}} r^2 g_{ab}(r)dr, \tag{2}$$

with r_shell the location of the first minima in $g_{ab}(r)$.

3. Determining Reaction Rates

The OH^- lifetimes (τ) were assessed with the stable states picture approach.[S23,S24] The Lionanalysis software[S25] was used to calculate the probability of a stable OH^- molecule to turn into a stable H_2O molecule within a timeshift Δt. For this analysis, a stable water molecule was defined by having 2 H atoms within 1.1 Å of O atom, while a stable hydroxide has only one H within 1.4 Å radius of O atom. The resulting correlation functions $C(\Delta t)$ of all MLMD simulations are shown in Fig. S1. The OH^- lifetimes are determined by fitting a double exponential, and τ is computed by taking the weighted average between the two

timescales;

$$C(\Delta t) = a \exp\left(-\frac{\Delta t}{\tau_1}\right) + (1-a)\exp\left(-\frac{\Delta t}{\tau_2}\right), \tag{3}$$

$$\tau = a\tau_1 + (1-a)\tau_2, \tag{4}$$

where τ_1 and τ_2 are the two timescales and a the weighting factor. The temperature dependence of τ can be fitted to the Arrhenius equation

$$\frac{1}{\tau} = A \exp\left(\frac{E_{\text{barr}}}{RT}\right), \tag{5}$$

where R is the universal gas constant, T the temperature and A the pre-exponential factor of the Arrhenius equation. Results of this assessment can be found in Table I of the main manuscript.

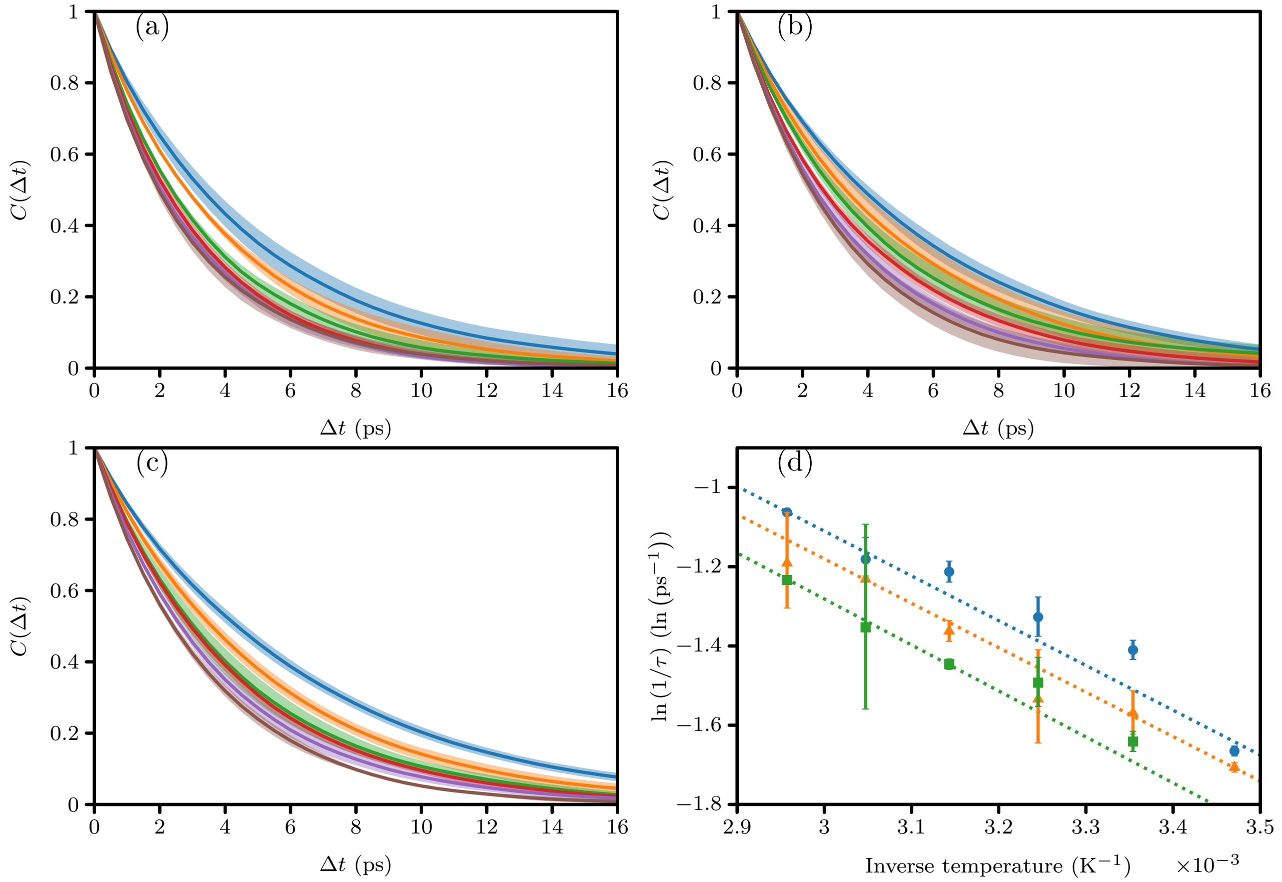

Figure S1: (a)-(c) Hydroxide lifetime correlation functions using ths stable state picture approach[S23,S24] for the light water, heavy water (D, deuterium), and very heavy water (T, tritium) simulations, respectively. Here, — is at 15 °C, — at 25 °C, — at 35 °C, — at 45 °C, — at 55 °C, and — at 65 °C. The shaded area indicates twice the standard deviation of the average simulation results at the same temperature. (d) The effective lifetime as function of inverse temperature. • are the results with H, ▲ the results of D, and ■ those of T. The Grotthuss transfer energy barriers are calculated from the temperature dependence of τ using the Arrhenius equation, of which the results are shown as dashed lines.

4. Transport Properties

The OH^- ions are tracked as a function of time and the self-diffusion coefficients are determined using the Einstein-Helfand relation.[S26,S27] Self-diffusion coefficients D_i are corrected for finite-size effects[S28–S30] using the Yeh-Hummer correction,

$$D_i = D_i^{\mathrm{MD}} + \frac{k_{\mathrm{B}}T\xi}{6\pi\eta L} = \lim_{t\to\infty} \frac{1}{6N_i t} \left\langle \sum_{j=1}^{N_i} \left(\mathbf{r}_{j,i}(t_0+t) - \mathbf{r}_{j,i}(t_0)\right)^2 \right\rangle + \frac{k_{\mathrm{B}}T\xi}{6\pi\eta L}, \quad (6)$$

where D_i is the self-diffusion coefficient of species i at the thermodynamic limit, N_i the number of particles of species i, and $\mathbf{r}_{j,i}$ indicates the atomic position of atom j of species i. t_0 and t are the time origin and time shift, respectively. The finite-size correction uses the experimentally determined mean shear viscosity η.[S31] The viscosities used for the Yeh-Hummer corrections are listed in Table S5. The box size is indicated by L and the dimensionless constant for cubic lattices $\xi = 2.837\,298$.[S28–S30,S30,S32,S33] The finite-size correction is the same for all species in the mixture. The electrical conductivity σ is computed using the Nernst-Einstein relation,[S34,S35]

$$\sigma = \frac{N_{\text{ions}} q^2}{V k_{\text{B}} T} \left(D_{\text{K}^+} + D_{\text{OH}^-} \right). \tag{7}$$

This depends on the total number of ion pairs N_{ions}, the electrical charge, and the finite-size corrected self-diffusion coefficient of the positive and negative ions, D_{K^+} and D_{OH^-}. This equation is valid for infinite dilute mixtures, as ion-ion correlations are neglected.[S36] While our simulations are performed close to the infinite dilution limit, direct electrical conductivity calculations of the Classical MD as well as 3 ns MLMD simulations are performed with the Einstein-Helfand equation,[S36,S37]

$$\sigma_{\text{D}} = \frac{N e^2}{V k_{\text{B}} T} \left(\Lambda_{\text{K}^+\text{K}^+} - 2\Lambda_{\text{K}^+\text{OH}^-} + \Lambda_{\text{OH}^-\text{OH}^-} \right), \tag{8}$$

$$\Lambda_{ij} = \frac{1}{6N} \lim_{t\to\infty} \frac{\text{d}}{\text{d}t} \left\langle \sum_{k=1}^{N_i} \sum_{l=1}^{N_j} \left[\mathbf{r}_i(t) - \mathbf{r}_i(0) \right] \cdot \left[\mathbf{r}_j(t) - \mathbf{r}_j(0) \right] \right\rangle. \tag{9}$$

were Λ_{ij} is the Onsager coefficient of molecule type i to type j. This method includes ion-ion correlation effects, but requires much longer simulation time and length scales for accurate results.[S36] This is because ion-ion correlations are small at low concentrations and there are no known finite-size corrections for Onsager coefficients.[S30] Direct electrical conductivities of the classical MD simulations computed with the Einstein-Helfands equation are listed

in Table S5 and for MLMD simulations in Table S6. The simulations settings for the 3 ns MLMD simulations are identical to the 1 ns MLMD simulations listed in Table S2 with 6 000 000 time steps.

The mean squared displacements of all MLMD and classical MD simulations are shown below: Fig. S2 illustrates the mean squared displacements (MSD) of the MLMD simulations with a hydrogen mass of 1 u, Fig. S3 the classical MD simulations, and Figs. S4 and S5 the MSDs of the MLMD simulations with increased hydrogen mass of 2, and 3 u, respectively.

Table S5: Calculated transport properties of KOH(aq). The self-diffusion is determined with Eq. (6) and the electrical conductivity with Eq. (7). Both system size depending self-diffusion as well as the self-diffusion in the thermodynamic limit are presented. The indicated shear viscosity values are derived using an experimental fit curve[S31] (MLMD simulations), or calculated from the simulation outputs using OCTP[S18](classical MD simulations). Direct electrical conductivities $\sigma_{\mathrm{D}}^{\mathrm{MD}}$ of the classical MD simulations inclulde ion-ion correlations and excluding finite-size effects by applying the Einstein-Helfand equation shown in Eq. (8). σ is the electrical conductivity as reported in the main document, computed with the Nernst-Einstein equation and corrected for finite-size effects. The values between parentheses are twice the standard deviation of the mean in the least-significant digits.

	Temperature (K)	288.15	298.15	308.15	318.15	328.15	338.15
**MLMD** $m_{\mathrm{H}} = 1\,\mathrm{u}$	$D_{\mathrm{K^+}}^{\mathrm{MD}}$ ($\times 10^{-9}\mathrm{m^2\,s^{-1}}$)	1.5(2)	1.8(2)	2.5(1)	2.5(3)	2.69(9)	3.4(2)
	$D_{\mathrm{OH^-}}^{\mathrm{MD}}$ ($\times 10^{-9}\mathrm{m^2\,s^{-1}}$)	2.7(3)	3.4(5)	3.6(5)	4.8(3)	4.7(7)	5.1(6)
	$\eta_{\mathrm{exp.}}$ (mPa s)	1.152	0.933	0.770	0.649	0.558	0.489
	$D_{\mathrm{K^+}}$ ($\times 10^{-9}\mathrm{m^2\,s^{-1}}$)	1.9(2)	2.3(2)	3.0(1)	3.2(3)	3.78(9)	4.3(2)
	$D_{\mathrm{OH^-}}$ ($\times 10^{-9}\mathrm{m^2\,s^{-1}}$)	3.1(3)	3.8(5)	4.2(5)	5.5(3)	5.5(7)	6.1(6)
	σ ($\mathrm{S\,m^{-1}}$)	9.6(5)	11(1)	13.1(9)	15.2(8)	16(1)	17(1)
**MLMD** $m_{\mathrm{D}} = 2\,\mathrm{u}$	$D_{\mathrm{K^+}}^{\mathrm{MD}}$ ($\times 10^{-9}\mathrm{m^2\,s^{-1}}$)	1.3(1)	1.6(3)	2.1(4)	2.4(3)	3.0(1)	3.1(3)
	$D_{\mathrm{OH^-}}^{\mathrm{MD}}$ ($\times 10^{-9}\mathrm{m^2\,s^{-1}}$)	2.5(3)	2.7(5)	3.3(4)	3.9(4)	4.3(9)	5.4(3)
	$\eta_{\mathrm{exp.}}$ (mPa s)	1.152	0.933	0.770	0.649	0.558	0.489
	$D_{\mathrm{K^+}}$ ($\times 10^{-9}\mathrm{m^2\,s^{-1}}$)	1.7(2)	2.1(3)	2.7(2)	3.1(3)	3.8(1)	4.0(2)
	$D_{\mathrm{OH^-}}$ ($\times 10^{-9}\mathrm{m^2\,s^{-1}}$)	2.9(3)	3.2(5)	3.9(4)	4.7(4)	5.1(9)	6.4(3)
	σ ($\mathrm{S\,m^{-1}}$)	8.9(7)	10.0(9)	11.8(9)	13.4(5)	15(1)	17.1(7)
**MLMD** $m_{\mathrm{T}} = 3\,\mathrm{u}$	$D_{\mathrm{K^+}}^{\mathrm{MD}}$ ($\times 10^{-9}\mathrm{m^2\,s^{-1}}$)	1.2(2)	1.7(1)	1.8(2)	2.1(2)	2.5(2)	2.9(2)
	$D_{\mathrm{OH^-}}^{\mathrm{MD}}$ ($\times 10^{-9}\mathrm{m^2\,s^{-1}}$)	2.6(2)	3.1(3)	3.4(3)	3.7(6)	4.0(5)	5.4(9)
	$\eta_{\mathrm{exp.}}$ (mPa s)	1.152	0.933	0.770	0.649	0.558	0.489
	$D_{\mathrm{K^+}}$ ($\times 10^{-9}\mathrm{m^2\,s^{-1}}$)	1.6(2)	2.1(1)	2.4(2)	2.8(2)	3.3(2)	3.8(2)
	$D_{\mathrm{OH^-}}$ ($\times 10^{-9}\mathrm{m^2\,s^{-1}}$)	2.9(2)	3.5(3)	3.9(3)	4.4(6)	4.9(5)	6.3(9)
	σ ($\mathrm{S\,m^{-1}}$)	8.9(5)	10.7(6)	11.5(5)	13(1)	13.8(8)	16(2)
**Classical MD**	$D_{\mathrm{K^+}}^{\mathrm{MD}}$ ($\times 10^{-9}\mathrm{m^2\,s^{-1}}$)	1.44(4)	1.79(4)	2.17(6)	2.60(4)	3.00(6)	3.52(4)
	$D_{\mathrm{OH^-}}^{\mathrm{MD}}$ ($\times 10^{-9}\mathrm{m^2\,s^{-1}}$)	0.77(2)	1.01(3)	1.28(4)	1.57(3)	1.90(4)	2.29(3)
	$\sigma_{\mathrm{D}}^{\mathrm{MD}}$ ($\mathrm{S\,m^{-1}}$)	3.7(2)	4.3(3)	5.3(1)	6.2(3)	6.7(3)	7.5(5)
	$\eta_{\mathrm{sim.}}$ (mPa s)	1.10(3)	0.87(2)	0.73(2)	0.61(3)	0.53(2)	0.45(2)
	$D_{\mathrm{K^+}}$ ($\times 10^{-9}\mathrm{m^2\,s^{-1}}$)	1.61(5)	2.01(4)	2.44(7)	2.93(5)	3.40(6)	4.00(4)
	$D_{\mathrm{OH^-}}$ ($\times 10^{-9}\mathrm{m^2\,s^{-1}}$)	0.95(2)	1.23(3)	1.55(4)	1.91(3)	2.30(4)	2.78(3)
	σ ($\mathrm{S\,m^{-1}}$)	5.0(1)	6.1(1)	7.3(1)	8.5(1)	9.6(1)	11.03(7)

Table S6: Calculated transport properties of KOH(aq) using 3 ns MLMD simulations. The superscript MD in the self-diffusion and electrical conductivity results are not corrected for finite-size effects, unlike the other results. The electrical conductivity results are computed with the Nernst-Einstein equation (σ and σ^{MD} with Eq. (7). The directly computed electrical conductivities determined with Eq. (8) are indicated with subscript D. The indicated shear viscosity values are derived using an experimental fit curve[S31] and used for the Yeh-Hummer[S29,S30] finite-size corrections. The values between parentheses are twice the standard deviation of the mean in the least-significant digits.

	Temperature (K)	288.15	298.15	308.15	318.15	328.15	338.15
**MLMD** $m_{\mathrm{H}} = 1\,\mathrm{u}$	$D^{\mathrm{MD}}_{\mathrm{K}^+}$ ($\times 10^{-9}\mathrm{m}^2\,\mathrm{s}^{-1}$)	1.7(1)	2.0(1)	2.20(3)	2.6(2)	3.2(2)	3.3(2)
	$D^{\mathrm{MD}}_{\mathrm{OH}^-}$ ($\times 10^{-9}\mathrm{m}^2\,\mathrm{s}^{-1}$)	3.0(3)	3.5(2)	3.8(4)	4.4(2)	5.4(5)	5.8(3)
	σ^{MD} ($\mathrm{S\,m}^{-1}$)	9.2(8)	10.2(5)	10.8(8)	12.4(4)	14(1)	14.8(8)
	$\sigma^{\mathrm{MD}}_{\mathrm{D}}$ ($\mathrm{S\,m}^{-1}$)	9(2)	10.1(7)	11(1)	12(1)	14(2)	15(1)
	$\eta_{\mathrm{exp.}}$ (mPa s)	1.152	0.933	0.770	0.649	0.558	0.489
	D_{K^+} ($\times 10^{-9}\mathrm{m}^2\,\mathrm{s}^{-1}$)	2.0(1)	2.4(1)	2.76(3)	3.3(2)	4.0(2)	4.3(2)
	D_{OH^-} ($\times 10^{-9}\mathrm{m}^2\,\mathrm{s}^{-1}$)	3.4(3)	3.9(2)	4.3(4)	5.1(2)	6.2(5)	6.7(3)
	σ ($\mathrm{S\,m}^{-1}$)	10.6(8)	11.9(5)	12.8(8)	14.7(4)	17(1)	17.9(8)

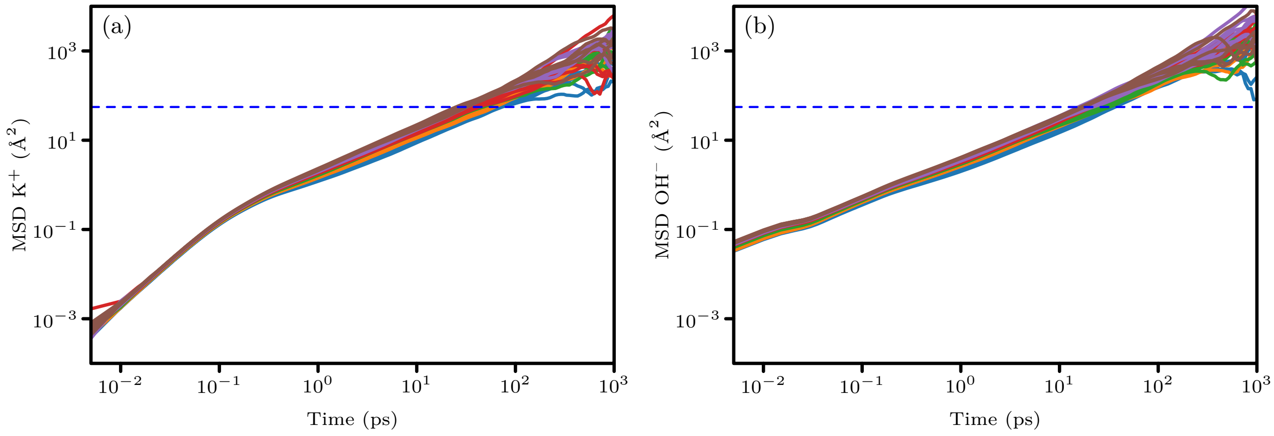

Figure S2: Mean squared displacements of the MLMD simulations as function of time for (a) K^+ and (b) OH^- ions. The colors indicate different temperatures; —— is at 15 °C, —— at 25 °C, —— at 35 °C, —— at 45 °C, —— at 55 °C, and —— at 65 °C. Half the box size squared is indicated with - - -. The calculated self-diffusion coefficients and electrical conductivity are listed in Table S5.

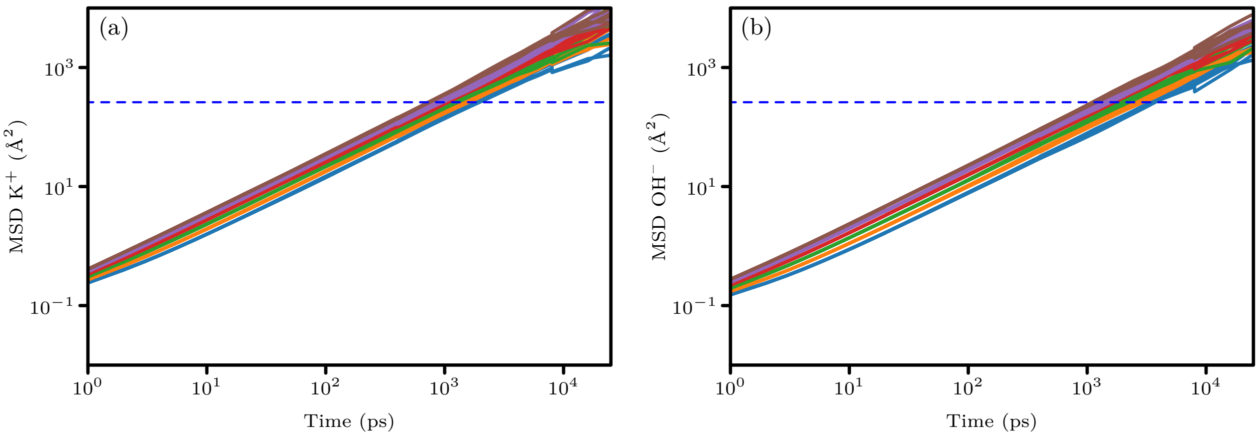

Figure S3: Mean squared displacements of the classical MD simulations as function of time for (a) K^+ and (b) OH^- ions. The colors indicate different temperatures; — is at 15 °C, — at 25 °C, — at 35 °C, — at 45 °C, — at 55 °C, and — at 65 °C. Half the box size squared is indicated with - - -. The calculated self-diffusion coefficients and electrical conductivity are listed in Table S5.

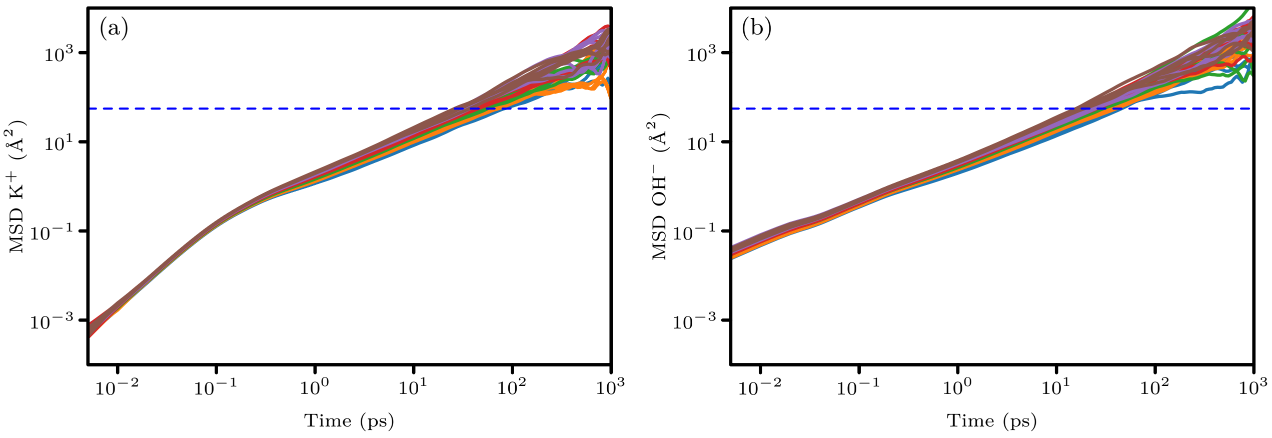

Figure S4: Mean squared displacements of the MLMD simulations of heavy water ($m_{\mathrm{D}} = 2\,\mathrm{u}$) as function of time for (a) K^+ and (b) OH^- ions. The colors indicate different temperatures; — is at 15 °C, — at 25 °C, — at 35 °C, — at 45 °C, — at 55 °C, and — at 65 °C. Half the box size squared is indicated with - - -. The calculated self-diffusion coefficients and electrical conductivity are listed in Table S5.

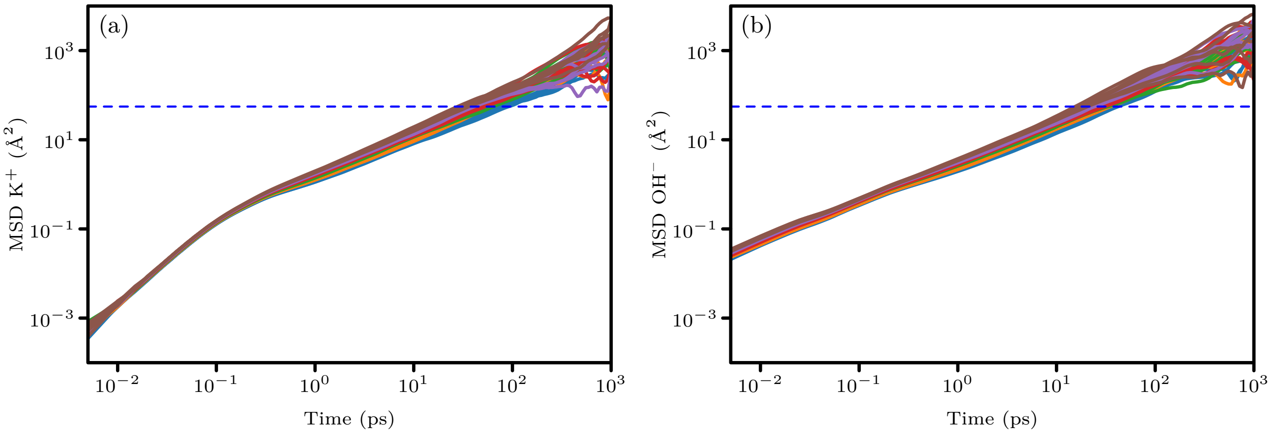

Figure S5: Mean squared displacements of the MLMD simulations of heavy water ($m_\mathrm{T} = 3\,\mathrm{u}$) as function of time for (a) K$^+$ and (b) OH$^-$ ions. The colors indicate different temperatures; — is at 15 °C, — at 25 °C, — at 35 °C, — at 45 °C, — at 55 °C, and — at 65 °C. Half the box size squared is indicated with - - -. The calculated self-diffusion coefficients and electrical conductivity are listed in Table S5.

5. Graph theory analysis

Trajectories have been analyzed using the GaTewAY graph-theoretical tool[S38–S40] to characterize the time-dependent hydrogen bonding patterns of OH^- in liquid water. These graph analyses identified the structural properties of the reactive and nonreactive configurations. Topological 2D-MolGraphs[S41] are defined at the atomic level of representation, with the vertices representing the atoms of the molecular system and the edges representing the bonds and interactions between the atoms. In this work, edges are associated to the O-H covalent bonds (water and OH^-) and to the O-H...O intermolecular hydrogen bonding interactions.[S40,S41] Other relationships between intermolecular interactions and graph edges can be found in previous works.[S39,S40] Hydrogen atoms are not included in vertices of a 2D-MolGraph, instead, their presence is known by directed edges that are associated to a hydrogen bond. The direction of the arc provides the knowledge of the donor and acceptor atoms in the H-Bond. With these definitions of the 2D-MolGraphs, the analysis of a given molecular dynamics simulation proceeds as follows:

1. Read the first snapshot 0 and define the associated 2D-MolGraph G_0,

2. Sequentially read the next snapshots i, one by one, calculate the associated 2D-MolGraph G_i,

3. At time step i, test if G_i is isomorphic to any other G_j already identified at previous snapshots $j < i$,

4. If yes, assign G_i to the set of conformations already identified; Else, assign G_i to the set of non-isomorphic conformations,

5. Continue with step $(i + 1)$ to read the subsequent snapshot(s).

Thus, a molecular dynamics simulation corresponds to a collection of non-isomorphic 2D-MolGraphs, with their time sequence being well-defined. Specifically, it captures the

temporal evolution of hydrogen bond formation and breaking, as well as covalent bond dynamics. This is in particular used to quantify the hydrogen bonding patterns of OH^- before and after the proton transfer reactions.

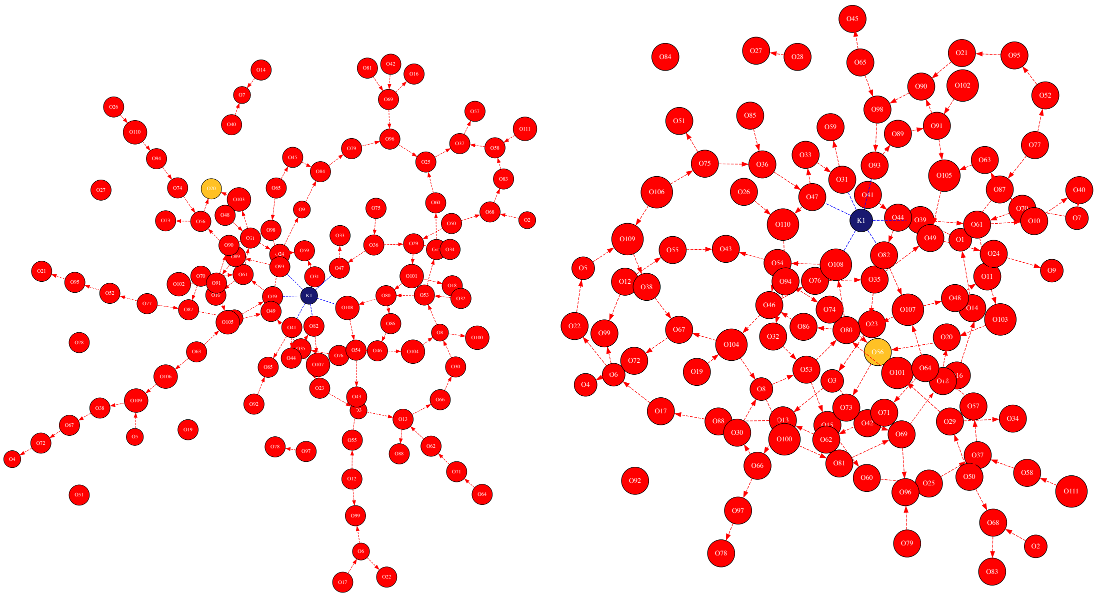

(a) (b)

Figure S6: Two consecutive 2D-MolGraphs provided by the GaTewAY tool. The oxygen in the OH^- ion is indicated as an orange vertex. (a) 2D-MolGraph captured at a stage prior to proton transfer. In this configuration, the oxygen atom in the OH^- is O20. (b) 2D-MolGraph after the proton transfer, where the oxygen atom in the OH^- switched to O56.

Figure S6a and Fig. S6b illustrate two 2D-MolGraphs provided by the GaTewAY tool (for chosen snapshots of the MD trajectories). Figure S6a presents a 2D-MolGraph captured at a stage prior to proton transfer. The OH^- is colored in orange in the graph through its vertex (O20). One can see that OH^- is involved in 2 hydrogen bonds (0 as donor and 2 as acceptor) shown with the 2 directed red dashed edges associated to the O20 vertex. After the proton transfer arising from the nearby water molecule which vertex is labeled O56, vertex O20 is now colored in red (see Fig. S6b) as this vertex is now associated with 2 hydrogen atoms covalently bonded and forming a water molecule, while the water with oxygen O56 (now in orange) becomes the OH^- species with only one O-H covalent bond. Subsequent to the proton transfer, water/vertex O20 is involved in 3 H-Bonds (1 as donor

and 2 as acceptor) as nicely seen with the 3 directed edges. In these 2D-MolGraphs, the potassium cation is associated with a dark blue vertex, and one can see dark dashed edges associated to that vertex and surrounding red vertices/water molecules. These represent the electrostatic interactions between K^+ and its first solvation shell of water molecules. The statistical and time-dependent knowledge of the solvation shell of K^+ can also easily be extracted from the analysis of the 2D-MolGraphs.[S42] Figures 3.c and 3.d in the main text show only a part of the 2D-MolGraphs provided by GaTewAY. The whole 2D-MolGraphs are shown in figures Fig. S7a and Fig. S7b.

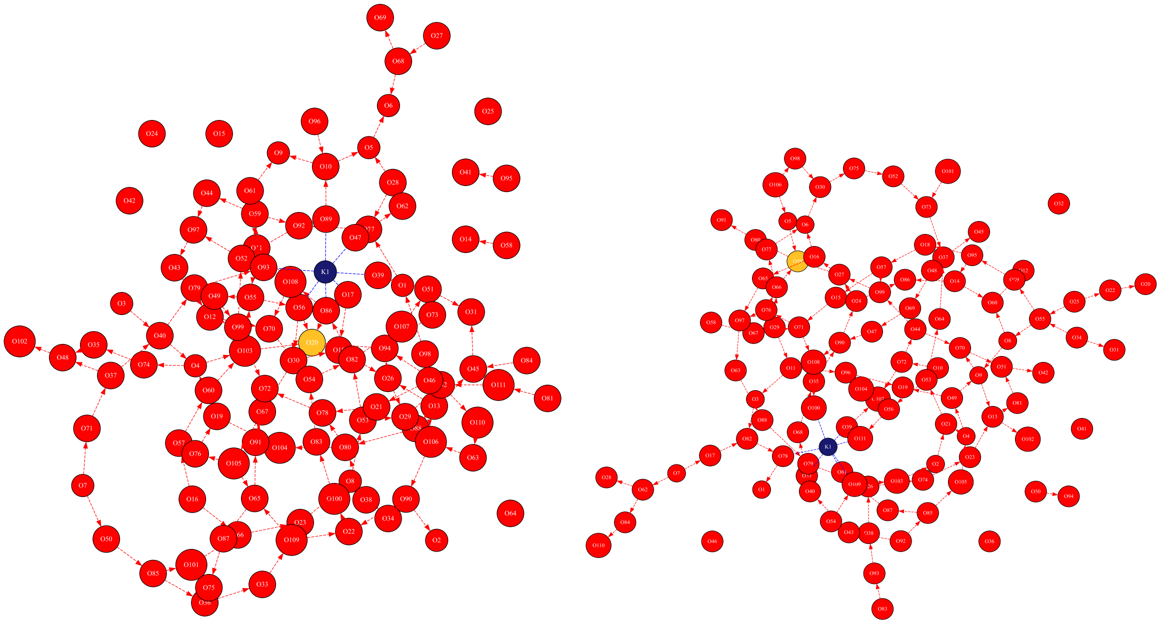

(a) (b)

Figure S7: Illustration of the origional 2D-MolGraphs corresponding to the sub-graphs in Fig. 3.c and 3.d in the main text. The figures in the main text show the relevant parts of the graphs (the hydration of the OH^-) in a decluttered manner. The location of the vertices in the main text are adjusted manually without changing the edges connecting these vertices.

References

(S1) Jinnouchi, R.; Lahnsteiner, J.; Karsai, F.; Kresse, G.; Bokdam, M. Phase Transitions of Hybrid Perovskites Simulated by Machine-Learning Force Fields Trained on the

Fly with Bayesian Inference. *Phys. Rev. Lett.* **2019**, *122*, 225701.

(S2) Jinnouchi, R.; Karsai, F.; Kresse, G. On-the-Fly Machine Learning Force Field Generation: Application to Melting Points. *Phys. Rev. B* **2019**, *100*, 014105.

(S3) Jinnouchi, R.; Miwa, K.; Karsai, F.; Kresse, G.; Asahi, R. On-the-Fly Active Learning of Interatomic Potentials for Large-Scale Atomistic Simulations. *J. Phys. Chem. Lett.* **2020**, *11*, 6946–6955.

(S4) Jinnouchi, R.; Karsai, F.; Kresse, G. Making Free-Energy Calculations Routine: Combining First Principles with Machine Learning. *Phys. Rev. B* **2020**, *101*, 060201.

(S5) Kresse, G.; Hafner, J. Ab Initio Molecular Dynamics for Liquid Metals. *Phys. Rev. B* **1993**, *47*, 558–561.

(S6) Kresse, G.; Furthmüller, J. Efficiency of Ab-Initio Total Energy Calculations for Metals and Semiconductors Using a Plane-Wave Basis Set. *Comput. Mater. Sci.* **1996**, *6*, 15–50.

(S7) Kresse, G.; Furthmüller, J. Efficient Iterative Schemes for Ab Initio Total-Energy Calculations Using a Plane-Wave Basis Set. *Phys. Rev. B* **1996**, *54*, 11169–11186.

(S8) Kresse, G.; Joubert, D. From Ultrasoft Pseudopotentials to the Projector Augmented-Wave Method. *Phys. Rev. B* **1999**, *59*, 1758–1775.

(S9) Hammer, B.; Hansen, L. B.; Nørskov, J. K. Improved Adsorption Energetics within Density-Functional Theory Using Revised Perdew-Burke-Ernzerhof Functionals. *Phys. Rev. B* **1999**, *59*, 7413–7421.

(S10) Perdew, J. P.; Burke, K.; Ernzerhof, M. Generalized Gradient Approximation Made Simple. *Phys. Rev. Lett.* **1996**, *77*, 3865–3868.

(S11) Grimme, S.; Antony, J.; Ehrlich, S.; Krieg, H. A Consistent and Accurate Ab Initio Parametrization of Density Functional Dispersion Correction (DFT-D) for the 94 Elements H-Pu. *J. Chem. Phys.* **2010**, *132*, 154104.

(S12) Grimme, S.; Ehrlich, S.; Goerigk, L. Effect of the Damping Function in Dispersion Corrected Density Functional Theory. *J. Comput. Chem.* **2011**, *32*, 1456–1465.

(S13) Gilliam, R. J.; Graydon, J. W.; Kirk, D. W.; Thorpe, S. J. A Review of Specific Conductivities of Potassium Hydroxide Solutions for Various Concentrations and Temperatures. *Int. J. Hydrog. Energy* **2007**, *32*, 359–364.

(S14) Padua, A.; Goloviznina, K.; Gong, Z. Fftool: XML Force Field Files. 2021.

(S15) Martínez, L.; Andrade, R.; Birgin, E. G.; Martínez, J. M. PACKMOL: A package for building initial configurations for molecular dynamics simulations. *J. Comput. Chem.* **2009**, *30*, 2157–2164.

(S16) Plimpton, S. Fast Parallel Algorithms for Short-Range Molecular Dynamics. *J. Comput. Phys.* **1995**, *117*, 1–19.

(S17) Thompson, A. P.; Aktulga, H. M.; Berger, R.; Bolintineanu, D. S.; Brown, W. M.; Crozier, P. S.; in 't Veld, P. J.; Kohlmeyer, A.; Moore, S. G.; Nguyen, T. D.; Shan, R.; Stevens, M. J.; Tranchida, J.; Trott, C.; Plimpton, S. J. LAMMPS - a Flexible Simulation Tool for Particle-Based Materials Modeling at the Atomic, Meso, and Continuum Scales. *Comput. Phys. Commun.* **2022**, *271*, 108171.

(S18) Jamali, S. H.; Wolff, L.; Becker, T. M.; de Groen, M.; Ramdin, M.; Hartkamp, R.; Bardow, A.; Vlugt, T. J. H.; Moultos, O. A. OCTP: A Tool for On-the-Fly Calculation of Transport Properties of Fluids with the Order-n Algorithm in LAMMPS. *J. Chem. Inf. Model.* **2019**, *59*, 1290–1294.

(S19) Abascal, J. L. F.; Vega, C. A General Purpose Model for the Condensed Phases of Water: TIP4P/2005. *J. Chem. Phys.* **2005**, *123*, 234505.

(S20) Habibi, P.; Rahbari, A.; Blazquez, S.; Vega, C.; Dey, P.; Vlugt, T. J. H.; Moultos, O. A. A New Force Field for OH^- for Computing Thermodynamic and Transport Properties of H_2 and O_2 in Aqueous NaOH and KOH Solutions. *J. Phys. Chem. B* **2022**, *126*, 9376–9387.

(S21) Borgis, D.; Assaraf, R.; Rotenberg, B.; Vuilleumier, R. Computation of Pair Distribution Functions and Three-Dimensional Densities with a Reduced Variance Principle. *Mol. Phys.* **2013**, *111*, 3486–3492.

(S22) Rotenberg, B. Use the Force! Reduced Variance Estimators for Densities, Radial Distribution Functions, and Local Mobilities in Molecular Simulations. *J. Chem. Phys.* **2020**, *153*, 150902.

(S23) Northrup, S. H.; Hynes, J. T. The Stable States Picture of Chemical Reactions. I. Formulation for Rate Constants and Initial Condition Effects. *J. Chem. Phys.* **1980**, *73*, 2700–2714.

(S24) Laage, D.; Hynes, J. T. On the Residence Time for Water in a Solute Hydration Shell: Application to Aqueous Halide Solutions. *J. Phys. Chem. B* **2008**, *112*, 7697–7701.

(S25) Hellström, M.; Ceriotti, M.; Behler, J. Nuclear Quantum Effects in Sodium Hydroxide Solutions from Neural Network Molecular Dynamics Simulations. *J. Phys. Chem. B* **2018**, *122*, 10158–10171.

(S26) Helfand, E. Transport Coefficients from Dissipation in a Canonical Ensemble. *Phys. Rev.* **1960**, *119*, 1–9.

(S27) Frenkel, D.; Smit, B. *Understanding Molecular Simulation: From Algorithms to Applications*, 3rd ed.; Academic Press: San Diego, USA, 2023.

(S28) Dünweg, B.; Kremer, K. Molecular Dynamics Simulation of a Polymer Chain in Solution. *J. Chem. Phys.* **1993**, *99*, 6983–6997.

(S29) Yeh, I.-C.; Hummer, G. System-Size Dependence of Diffusion Coefficients and Viscosities from Molecular Dynamics Simulations with Periodic Boundary Conditions. *J. Phys. Chem. B* **2004**, *108*, 15873–15879.

(S30) Celebi, A. T.; Jamali, S. H.; Bardow, A.; Vlugt, T. J. H.; Moultos, O. A. Finite-Size Effects of Diffusion Coefficients Computed from Molecular Dynamics: A Review of What We Have Learned so Far. *Mol. Simul.* **2021**, *47*, 831–845.

(S31) Guo, Y.-j.; Xu, H.-b.; Guo, F.; Zheng, S.-l.; Zhang, Y. Density and Viscosity of Aqueous Solution of K_2CrO_4/KOH Mixed Electrolytes. *Trans. Nonferrous Met. Soc. China.* **2010**, *20*, s32–s36.

(S32) Moultos, O. A.; Zhang, Y.; Tsimpanogiannis, I. N.; Economou, I. G.; Maginn, E. J. System-Size Corrections for Self-Diffusion Coefficients Calculated from Molecular Dynamics Simulations: The Case of CO_2, n-Alkanes, and Poly(Ethylene Glycol) Dimethyl Ethers. *J. Chem. Phys.* **2016**, *145*, 074109.

(S33) Jamali, S. H.; Hartkamp, R.; Bardas, C.; Söhl, J.; Vlugt, T. J. H.; Moultos, O. A. Shear Viscosity Computed from the Finite-Size Effects of Self-Diffusivity in Equilibrium Molecular Dynamics. *J. Chem. Theory Comput.* **2018**, *14*, 5959–5968.

(S34) Hu, Z.; Jiang, J. Assessment of Biomolecular Force Fields for Molecular Dynamics Simulations in a Protein Crystal. *J. Comput. Chem.* **2010**, *31*, 371–380.

(S35) Zhang, Y.; Maginn, E. J. Direct Correlation between Ionic Liquid Transport Properties and Ion Pair Lifetimes: A Molecular Dynamics Study. *J. Phys. Chem. Lett.* **2015**, *6*, 700–705.

(S36) Blazquez, S.; Abascal, J. L. F.; Lagerweij, J.; Habibi, P.; Dey, P.; Vlugt, T. J. H.; Moultos, O. A.; Vega, C. Computation of Electrical Conductivities of Aqueous Electrolyte Solutions: Two Surfaces, One Property. *J. Chem. Theory Comput.* **2023**, *19*, 5380–5393.

(S37) Gullbrekken, Ø.; Røe, I. T.; Selbach, S. M.; Schnell, S. K. Charge Transport in Water–NaCl Electrolytes with Molecular Dynamics Simulations. *J. Phys. Chem. B* **2023**, *127*, 2729–2738.

(S38) Bougueroua, S.; Quessette, F.; Barth, D.; Gaigeot, M.-P. GaTewAY : Graph Theory-Based Software for Automatic Analysis of Molecular Conformers Generated over Time. 2022.

(S39) Hashemi, A.; Bougueroua, S.; Gaigeot, M.-P.; Pidko, E. A. ReNeGate: A Reaction Network Graph-Theoretical Tool for Automated Mechanistic Studies in Computational Homogeneous Catalysis. *J. Chem. Theory Comput.* **2022**, *18*, 7470–7482.

(S40) Bougueroua, S.; Aboulfath, Y.; Cimas, A.; Hashemi, A.; A. Pidko, E.; Barth, D.; Gaigeot, M.-P. Topological graphs: a review of some of our achievements and perspectives in physical chemistry and homogeneous catalysis. *Comptes Rendus. Chimie* **2024**, *27*, 1–23.

(S41) Bougueroua, S.; Spezia, R.; Pezzotti, S.; Vial, S.; Quessette, F.; Barth, D.; Gaigeot, M.-P. Graph Theory for Automatic Structural Recognition in Molecular Dynamics Simulations. *J. Chem. Phys.* **2018**, *149*, 184102.

(S42) Bougueroua, S.; Aboulfath, Y.; Barth, D.; Gaigeot, M.-P. Algorithmic Graph Theory for Post-Processing Molecular Dynamics Trajectories. *Mol. Phys.* **2023**, *121*, e2162456.
